# An Intelligent Interactive Management Platform for Rheumatoid Arthritis Care: Real-World Observational Study

**DOI:** 10.2196/90784

**Published:** 2026-04-02

**Authors:** Ziyun Zhang, Xinyue Zhang, Li Zhang, Mingyan Du, Lijuan Zhang, Siyu Yang, Xiaoli Cai, Shengchao Hou

**Affiliations:** 1 Department of Nursing, Tongji Hospital, Tongji Medical College, Huazhong University of Science and Technology, 1095 Jiefang Road, Qiaokou District, Wuhan, Hubei, 430030, China, 86 18207121823; 2 Department of Rheumatology and Immunology, Tongji Hospital, Tongji Medical College, Huazhong University of Science and Technology, Wuhan, Hubei, China; 3 School of Nursing, Tongji Medical College, Huazhong University of Science and Technology, Wuhan, Hubei, China; 4 Library, Tongji Hospital, Tongji Medical College, Huazhong University of Science and Technology, Wuhan, Hubei, China

**Keywords:** rheumatoid arthritis, intelligent interactive platform, disease activity, medication adherence, patient satisfaction, artificial intelligence, AI

## Abstract

**Background:**

Effective postdischarge management is essential for maintaining disease control and improving long-term outcomes in rheumatoid arthritis (RA). Digital health technologies, particularly intelligent management platforms, provide new opportunities for continuous care and self-management in real-world settings.

**Objective:**

This study aimed to develop a nurse-led artificial intelligence (AI)–assisted interactive management platform for postdischarge RA care and examine its association with patient outcomes in a real-world clinical setting.

**Methods:**

A single-center, multicampus, real-world observational study with a quasi-experimental framework was conducted at Tongji Hospital between November 2024 and October 2025. Participants were allocated to either a platform-based management group (campus 1; n=184) or a comparison group receiving routine postdischarge care (campus 2; n=157). The platform enabled remote monitoring through a smartphone app, which collected information on joint symptoms, fatigue, medication adherence, laboratory results, and emotional status, with data uploaded to a secure cloud server. The AI system analyzed data in real time and alerted health care providers to abnormalities. Nurses and health coaches delivered personalized education and interventions. Primary outcomes were changes in Disease Activity Score in 28 Joints and Health Assessment Questionnaire–II scores at discharge and the 6-month follow-up. Secondary outcomes included medication adherence measured as the proportion of prescribed doses taken and patient satisfaction assessed via a 5-point Likert-scale questionnaire at 6 months.

**Results:**

A total of 341 patients with RA completed the 6-month follow-up. Disease Activity Score in 28 Joints values decreased significantly in the platform-based management group at 6 months (mean 5.04, SD 1.38 at discharge to mean 4.27, SD 1.20 at 6 months; *P*<.001), showing a greater reduction than in the comparison group (mean 4.86, SD 1.19 at discharge to mean 4.59, SD 1.36 at 6 months; within-group *P*=.03; between-group *P*=.02). Health Assessment Questionnaire–II scores improved significantly in the platform-based management group (mean 2.45, SD 0.75 at discharge to mean 1.67, SD 0.56 at 6 months; *P*<.001), while the comparison group showed no significant change (*P*=.12; between-group *P*<.001). Medication adherence was higher in the platform-based group (166/184, 90.2% vs 125/157, 79.6%; *χ_1_*^2^=7.6; *P*=.006). Overall health care satisfaction was also higher in the platform-based group (176/184, 95.7% vs 120/157, 76.4%; *χ_1_*^2^=27.8; *P*<.001), with a greater proportion reporting to be “very satisfied” in this group (150/184, 81.5% vs 59/157, 37.6%).

**Conclusions:**

The intelligent interactive management platform markedly enhanced disease control, functional status, medication adherence, and satisfaction among patients with RA after discharge. This nurse-led, AI-assisted system represents a promising model for improving outpatient RA management.

## Introduction

Rheumatoid arthritis (RA) is a chronic systemic autoimmune disease with a global prevalence of approximately 1% to 2% [1]. It predominantly affects middle-aged and older adults, with higher prevalence among women aged 40 to 60 years [1,2]. RA is frequently complicated by extra-articular manifestations, including cardiovascular disease, pulmonary dysfunction, neurological involvement, gastrointestinal disturbances, and malnutrition [3]. Although no curative therapy exists, timely and standardized treatment can effectively control disease activity, relieve symptoms, and delay disability [4]. Long-term management commonly involves pharmacological therapy, intra-articular injections, physical rehabilitation, regular follow-up visits, and surgical intervention when indicated [5]. Nevertheless, despite ongoing treatment, many patients experience varying degrees of joint deformity and functional impairment, substantially limiting their daily activities and compromising overall well-being [6].

Given the chronic and progressive nature of RA, effective self-management is essential for maintaining patients’ quality of life and delaying disease progression [7]. Conventional nursing models primarily focus on inpatient care, whereas postdischarge management often relies heavily on patient self-management, leading to suboptimal outcomes [8]. Successful RA management requires patients to develop both the confidence and the necessary skills to manage their condition independently [9]. Key components of self-management include self-monitoring of symptoms and disease flares, setting achievable goals, adopting behavioral modifications, adhering to medication, adjusting physical activity levels, and enhancing problem-solving abilities [3]. Robust self-management practices have been shown to improve adherence, reduce disease activity, and slow progression of RA [10,11]. Therefore, innovative and sustainable strategies are needed to enhance postdischarge self-management and continuity of care.

The rapid proliferation of mobile technologies has transformed health service delivery worldwide [12]. As mobile apps become increasingly embedded in routine activities, the delivery of health care through digital platforms has emerged as an inevitable evolution in line with technological advancement [13]. In the context of RA management, mobile health (mHealth) technologies, including smartphones, wearable devices, and disease-specific apps, are playing an increasingly pivotal role [12,14]. These tools enable real-time health data collection, thereby facilitating early detection of disease fluctuations. They empower both patients and health professionals by enhancing disease monitoring, improving medication adherence, and enhancing timely personalized interventions [15]. Such innovations improve the efficiency of disease management by reducing unnecessary hospital visits and optimizing health care resource allocation. Furthermore, instant feedback mechanisms reinforce patients’ self-management capabilities and promote active engagement in their own care [16].

Building on our institution’s “internet hospital” infrastructure and standardized RA management protocols, we developed an intelligent, nurse-led interactive management platform to support postdischarge care for patients with RA. This study aimed to evaluate the real-world effectiveness of this platform by comparing patients managed through platform-based follow-up to those receiving routine postdischarge care across different hospital campuses under unified clinical standards.

## Methods

### Study Design and Population

This study used a single-center, multicampus observational design with a quasi-experimental framework conducted at the Department of Rheumatology and Immunology, Tongji Hospital, between November 2024 and October 2025. Tongji Hospital consists of multiple hospital campuses operating under unified clinical guidelines and standardized treatment protocols for RA. An intelligent interactive follow-up platform was first implemented at campus 1 as part of routine postdischarge care, whereas campus 2 had not yet adopted the platform at the time of the study. This natural difference in platform availability allowed for the formation of a platform-based management group (campus 1) and a comparison group receiving usual postdischarge care (campus 2) without individual-level assignment by the research team. Eligible participants were adults (aged ≥18 years) diagnosed with RA according to the 2010 American College of Rheumatology and European Alliance of Associations for Rheumatology classification criteria, receiving standardized RA therapy, and capable of using a smartphone or having a caregiver assist with platform access. Exclusion criteria included inability to comply with follow-up, severe psychiatric or cognitive disorders, visual or hearing impairment precluding platform use, severe systemic comorbidities (eg, advanced cardiac, hepatic, or renal disease), or discontinuation of RA treatment during the follow-up period.

### Group Allocation and Blinding

Participants were allocated to study groups based on hospital campus rather than through randomization. Patients discharged from campus 1 were included in the platform-based management group, whereas those discharged from campus 2 constituted the comparison group receiving routine postdischarge care. This campus-based allocation reflected real-world clinical implementation and helped avoid ethical concerns related to unequal access to a newly introduced digital management platform. Given the nature of the platform-based management model, blinding of patients and health care providers was not feasible. However, outcome assessors and data analysts were blinded to group allocation to reduce assessment and analysis bias.

### Technical Design and Platform-Based Management Model

#### Platform Overview

The intelligent interactive platform for RA is a multidisciplinary, nurse-led, and patient-centered digital health management system established within the smart health care ecosystem of Tongji Hospital. Designed to enhance routine outpatient care, the platform incorporates artificial intelligence (AI) and is operated by a specialized team of rheumatology nurses, with additional support from health coaches trained in rheumatology, nutrition, and psychological support.

The primary objective of the platform is to extend continuous care beyond hospital discharge. Timely responses to self-reported symptoms and system-generated alerts support individualized guidance and facilitate timely clinical decision-making during routine follow-up. It also facilitates complication monitoring, self-care education, and psychological support, helping patients manage their condition more effectively during home-based recovery. Integrated communication tools ensure that patients have continuous access to their care team.

#### Technical Architecture and Core Functions

##### Overview

**Figure 1.**
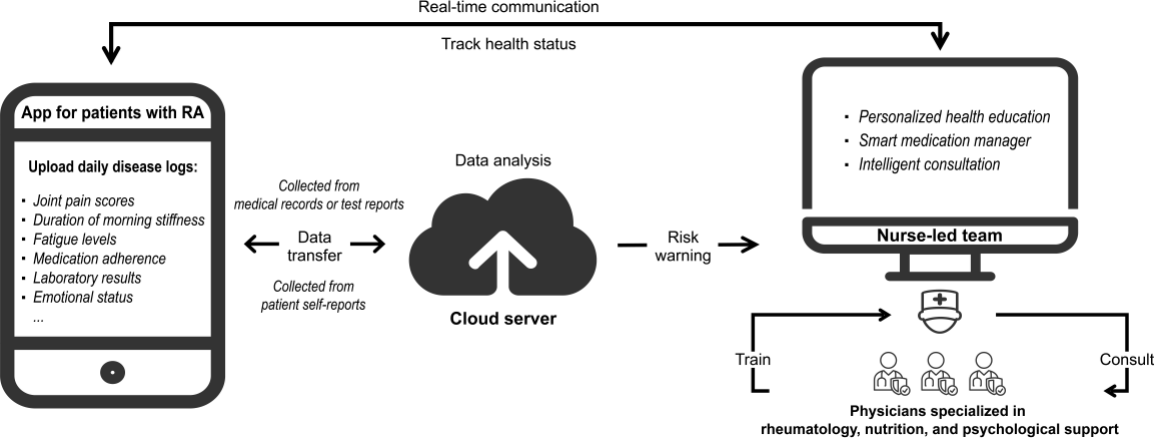
Framework of the intelligent interactive platform for rheumatoid arthritis (RA).

The platform was developed collaboratively by the hospital’s Information Center and technology partners. Its architecture comprises 3 core components: a patient-facing mobile app, a web-based dashboard for nurses and health coaches, and a cloud-based AI data processing system (Figure 1).

##### Data Input and Processing

Patients use the mobile app to submit health logs either daily or as needed based on their symptom fluctuations. These logs include joint pain scores measured using a visual analog scale from 1 to 100, duration of morning stiffness, fatigue levels, medication adherence, laboratory results, emotional status, and sleep quality. While the “Smart Medication Manager” provides automated daily notifications for drug intake, other health logs are recorded voluntarily by patients without daily reminders to avoid excessive burden. All data are securely encrypted and transmitted to the cloud server. For image-based data, such as medical records or test reports, a deep learning–based optical character recognition system automatically extracts key information, which is manually verified and structured into the database.

##### AI-Driven Clinical Decision Support

An embedded AI engine continuously analyzes patient data in real time. Unlike traditional rule-based systems that rely on static, predefined thresholds, our AI engine uses a multilayered approach. A deep learning–based optical character recognition system processes unstructured data from patient-uploaded images (eg, external laboratory reports). In addition, the engine uses longitudinal data processing to monitor trends in disease activity and medication adherence over time. When abnormalities are detected, such as elevated disease activity, decreased medication adherence, or signs of relapse, the system immediately generates alerts for the clinical care team through the dashboard even if individual parameters have not yet exceeded traditional laboratory triggers. Nurses and health coaches can then respond promptly via the app, providing timely follow-up support and facilitating triage for further outpatient or inpatient care if necessary (Multimedia Appendix 1).

##### Personalized Education and Self-Management Support

Beyond reactive support, the platform provides proactive, personalized health education. On the basis of individual patient assessments and preferences, the care team delivers guidance on diet, exercise, and psychological well-being. This is facilitated by an integrated intelligent education module that pushes customized content to patients (Multimedia Appendix 2). A “Smart Medication Manager” feature allows patients to log their prescribed oral medications and receive automated reminders to enhance adherence (Multimedia Appendix 3). Furthermore, an “Intelligent Consultation” module serves as a knowledge base for frequently asked questions related to RA care, covering topics such as appointments, insurance, follow-ups, and common rheumatological concerns, thereby improving accessibility and potentially reducing unnecessary clinic visits (Multimedia Appendix 4).

### Pilot Feasibility Assessment

Prior to the main study, an internal, unpublished pilot test was conducted to evaluate the platform’s feasibility and support its user-centered design. The pilot involved 25 patients with RA over a 4-week period. During this phase, the platform demonstrated high levels of engagement. Specifically, the patient self-assessment completion rate rose from 68.0% (17/25) to 88.0% (22/25). To ensure that the intervention was not solely investigator driven, we collected qualitative feedback from participants via telephone interviews and structured satisfaction surveys. Users identified several areas for improvement, such as the need for a more intuitive user interface for joint symptom logging and more flexible medication reminder settings. On the basis of the feedback, the platform underwent iterative refinements, including optimizing user interface responsiveness and streamlining the data entry process before the formal study commenced. While traditional clinical measures such as inflammatory markers and disease activity scores did not show statistically significant changes in the short-term pilot, promising improvements were observed in behavioral outcomes, including follow-up completion rates, platform log-in frequency, and consultation response times, indicating good usability and feasibility for real-world implementation.

### Data Collection and Study Procedure

Between November 7, 2024, and April 20, 2025, eligible patients with RA who were clinically stable and scheduled for discharge were consecutively recruited. Baseline data were collected prior to discharge. Participants were allocated to study groups based on hospital campus, reflecting routine clinical implementation of the intelligent interactive platform. All participants were followed up on for 6 months after enrollment, and the final follow-up assessment was completed on October 20, 2025.

During hospitalization, patients in both groups received standardized nursing care and health education in accordance with unified clinical protocols across campuses. This included RA-related disease education, psychological support, medication management, symptom monitoring, rehabilitation guidance, complication prevention, routine follow-up planning, and discharge counseling.

After discharge, patients in the comparison group received routine postdischarge follow-up care, which consisted of periodic assessments of disease status, emotional well-being, medication adherence, and rehabilitation activities. Follow-up was conducted using conventional approaches, including scheduled outpatient visits, telephone consultations, or home visits, during which health care professionals provided individualized guidance and addressed patient concerns as needed.

In addition to routine care, patients in the platform-based management group were supported through the platform. One to 2 days prior to discharge, designated rheumatology nurses introduced the platform to eligible patients using standardized instructions and obtained written informed consent. Nurses then provided individualized, hands-on guidance on platform access, account setup, and creation of a personal health record. Patients were guided through key functional modules to ensure independent use after discharge. For patients without access to a smartphone or those unfamiliar with mobile apps, family members or caregivers were instructed on how to operate the platform on the patient’s behalf to support ongoing disease management.

### Outcome Measures

#### Overview

Primary outcomes were disease activity and functional status assessments. Secondary outcomes included medication adherence and health care satisfaction. These outcomes were assessed to examine differences between groups rather than to evaluate causal effects of the platform. Disease activity and functional status were evaluated both at discharge and the 6-month follow-up, whereas secondary outcomes were assessed only at 6 months as many patients were treatment naive at baseline or satisfaction could not be measured until follow-up.

#### Disease Activity

The Disease Activity Score in 28 Joints (DAS28) was used to assess RA disease activity [17]. It combines a count of 28 swollen and tender joints, a laboratory marker of inflammation (either C-reactive protein or erythrocyte sedimentation rate), and a patient global health assessment. Higher DAS28 scores indicate more severe disease activity.

#### Functional Status

Functional disability was measured using the Health Assessment Questionnaire–II (HAQ-II) [18]. The HAQ-II is a validated instrument that assesses difficulty in performing activities of daily living across 8 domains (dressing, arising, eating, walking, hygiene, reach, grip, and common activities). It contains 10 items, each scored on a scale from 0 to 3 (0=“without difficulty”; 3=“unable to do”). The total score is the average of the category scores, with higher scores indicating greater functional impairment. The HAQ-II showed good internal consistency in this study (Cronbach α=0.83), consistent with its original validation (Cronbach α=0.88) [18].

#### Medication Adherence

Medication adherence was assessed based on participants’ self-reported medication intake during the follow-up period. Adherence was operationally defined as the proportion of prescribed doses reportedly taken. Consistent with commonly used adherence definitions in chronic disease management, participants were classified as having good adherence if they reported taking 85% or more of the prescribed medication regimen and poor adherence otherwise [19].

#### Health Care Satisfaction

Patient satisfaction with care was assessed using a department-developed questionnaire. Responses were recorded on a 5-point Likert scale (“very satisfied,” “satisfied,” “neutral,” “dissatisfied,” and “very dissatisfied”). The satisfaction rate was calculated as the percentage of participants reporting being “very satisfied” or “satisfied” among the total respondents. The scale’s content validity index was 0.92 as evaluated by a panel of 5 rheumatology experts, and the questionnaire demonstrated good reliability in this study (Cronbach α=0.86).

### Data Analysis

Statistical analyses were performed using R (version 4.4.1; R Foundation for Statistical Computing). Continuous variables were described as means and SDs for normally distributed data or medians and IQRs for nonnormally distributed data. Normality was assessed using the Shapiro-Wilk test. Variance homogeneity was evaluated using the Levene test. Between-group comparisons were performed using 2-tailed Student *t* test or Welch *t* test for normally distributed variables and the Mann-Whitney *U* test (Wilcoxon rank-sum test) for nonnormally distributed variables. Categorical variables were compared using the chi-square test or Fisher exact test, as appropriate. All *P* values were 2 sided and are reported as exact values in accordance with JMIR Publications’ statistical reporting guidelines.

### Ethical Considerations

This study was approved by the Medical Ethics Committee of Tongji Hospital, Tongji Medical College of Huazhong University of Science and Technology (TJ-IRB202411023). The study was conducted in accordance with the Declaration of Helsinki. Participation was voluntary, and written informed consent was obtained from all participants prior to enrollment. Participants retained the right to withdraw from the study at any time without penalty or explanation. All collected data were deidentified and stored securely in compliance with the hospital’s scientific research data management standards. Deidentification was performed using a key-encoded system, with reidentification keys securely stored by the study team. No financial incentives were offered to participants. The trial was prospectively registered at the Chinese Clinical Trial Registry (ChiCTR2500115743). This study is reported in accordance with the STROBE (Strengthening the Reporting of Observational Studies in Epidemiology) guidelines (Checklist 1).

## Results

### Demographic Characteristics

Between November 7, 2024, and April 20, 2025, a total of 459 patients with RA who met the inclusion criteria were identified at our hospital. Of these 459 patients, 401 (87.4%) consented to participate in the study, including 206 (44.9%) from campus 1 (platform-based management group) and 195 (42.5%) from campus 2 (comparison group). Following baseline assessment, 10.2% (21/206) of the patients from campus 1 and 20.0% (39/195) of the patients from campus 2 were lost to follow-up. The higher retention rate in the platform-based group may reflect the impact of continuous digital engagement. Ultimately, 89.3% (184/206) of the patients from campus 1 and 80.5% (157/195) of the patients from campus 2 completed the 6-month follow-up, resulting in a total of 341 (74.3%) participants included in the final analysis.

The baseline characteristics of the participants are summarized in Table 1. The mean age of the participants was 57.41 (SD 13.45) years in the platform-based management group and 55.47 (SD 13.57) years in the comparison group. Female individuals comprised most of both groups, accounting for 76.1% (140/184) and 78.3% (123/157) in the campus 1 and campus 2 groups, respectively. RA duration was similar between the 2 groups (median 4, IQR 2-12 years vs median 4, IQR 2-10 years). Educational levels, residence, smoking and alcohol consumption, BMI, RA disease duration, comorbidities including hypertension and diabetes, and RA-related complications did not differ significantly between the groups.

**Table 1.** Demographic characteristics of the participants (N=341).

Characteristic	Platform-based management group (n=184)	Comparison group (n=157)	*χ^2^* (*df*)	*P* value
Age (years)^a^, mean (SD)	57.41 (13.45)	55.47 (13.57)		.19
Sex, n (%)			0.133 (1)^b^	.72
Male	44 (23.9)	34 (21.7)		
Female	140 (76.1)	123 (78.3)		
Educational level, n (%)			5.324 (3)^b^	.15
Primary school	42 (22.8)	44 (28.0)		
Lower secondary school	71 (38.6)	56 (35.7)		
Upper secondary school	45 (24.5)	43 (27.4)		
Tertiary education	26 (14.1)	11 (7.0)		
Residence, n (%)			1.511 (1)^b^	.22
Rural	121 (65.8)	93 (59.2)		
Urban	63 (34.2)	64 (40.8)		
RA^c^ disease duration (years)^d^, median (IQR)	4 (2-12)	4 (2-10)		.54
BMI (kg/m^2^)^d^, median (IQR)	20.69 (19.33-22.79)	20.58 (19.23-22.77)		.78
Smoking, n (%)			0.030 (1)^b^	>.99
Yes	20 (10.9)	18 (11.5)		
No	164 (89.1)	139 (88.5)		
Alcohol consumption, n (%)			0.139 (1)^b^	.83
Yes	26 (14.1)	20 (12.7)		
No	158 (85.9)	137 (87.3)		
Hypertension, n (%)			3.269 (1)^b^	.07
Yes	61 (33.2)	38 (24.2)		
No	123 (66.8)	119 (75.8)		
Diabetes, n (%)			1.319 (1)^b^	.25
Yes	39 (21.2)	26 (16.6)		
No	145 (78.8)	131 (83.4)		
RA-related complications^e^, n (%)			0.052 (1)^b^	.89
Yes	95 (51.6)	79 (50.3)		
No	89 (48.4)	78 (49.7)		

a Student *t* test. Age: *t*_*339*_=1.321.

b Chi-square test.

c RA: rheumatoid arthritis.

d Mann-Whitney *U* test (Wilcoxon rank-sum test). RA disease duration: *W*=15,003. BMI: *W*=14,695.

e Primarily comprising interstitial lung disease, osteoporosis, and femoral head avascular necrosis.

### Changes in DAS28 Scores

Changes in disease activity over the 6-month follow-up are shown in Table 2. In the comparison group, the mean DAS28 score decreased from 4.86 (SD 1.19) at discharge to 4.59 (SD 1.36) at 6 months, with a statistically significant within-group change (*P*=.03). In the platform-based management group, the mean DAS28 score decreased from 5.04 (SD 1.38) at discharge to 4.27 (SD 1.20) at 6 months, with a larger within-group change (*P*<.001). Between-group comparison at 6 months indicated a significantly greater reduction in DAS28 scores in the platform-based management group than in the comparison group (*P*=.02).

**Table 2.** Disease Activity Score in 28 Joints values of the participants. *t_339_*=–1.293 and *P*=.20 at discharge and *t_339_*=2.286 and *P*=.02 at the 6-month follow-up. Disease Activity Score in 28 joints: range 0-9.4; higher scores indicate more activity.

Group	At discharge, mean (SD)	6-month follow-up, mean (SD)	Paired *t* test (*df*)	*P* value
Comparison group	4.86 (1.19)	4.59 (1.36)	2.172 (156)	.03
Platform-based management group	5.04 (1.38)	4.27 (1.20)	7.404 (183)	<.001

### Changes in HAQ-II Scores

At discharge, HAQ-II scores were comparable between the comparison group and the platform-based management group (*P*=.79; Table 3). At the 6-month follow-up, the comparison group showed no significant change from baseline (*P*=.12), whereas the platform-based management group exhibited a significant reduction (*P*<.001). Between-group comparison at 6 months indicated a significantly greater improvement in functional status in the platform-based management group than in the comparison group (*P*<.001).

**Table 3.** Health Assessment Questionnaire–II scores of the participants. *t*_339_=–1.293 and *P*=.20 at discharge and *t*_339_=2.286 and *P*=.02 at the 6-month follow-up. Disease Activity Score in 28 joints: range 0-9.4; higher scores indicate more activity.

Group	At discharge, mean (SD)	6-month follow-up, mean (SD)	Paired *t* test (*df*)	*P* value
Comparison group	2.47 (0.60)	2.39 (0.58)	1.565 (156)	.12
Platform-based management group	2.45 (0.75)	1.67 (0.56)	14.529 (183)	<.001

### Changes in Medication Adherence

On the basis of the predefined adherence criterion (≥85% of prescribed doses reportedly taken), 90.2% (166/184) of the participants in the platform-based management group exhibited good adherence compared with 79.6% (125/157) in the comparison group (*χ_1_*^2^=7.6; *P*=.006) at the 6-month follow-up.

### Changes in Health Care Satisfaction

Table 4 presents the health care satisfaction levels of participants in both groups. The overall satisfaction rates were 76.4% (120/157) in the comparison group and 95.7% (176/184) in the platform-based management group (*P*<.001). Notably, a greater proportion of participants in the platform-based management group reported being very satisfied compared to the comparison group (150/184, 81.5% vs 59/157, 37.6%, respectively).

**Table 4.** Health care satisfaction of the participants.

Group	Very satisfied, n (%)	Satisfied, n (%)	Neutral, n (%)	Dissatisfied, n (%)	Very dissatisfied, n (%)	Satisfaction rate (participants who were very satisfied or satisfied), n (%)^a^
Comparison group (n=157)	59 (37.6)	61 (38.9)	24 (15.3)	13 (8.3)	0 (0.0)	120 (76.4)
Platform-based management group (n=184)	150 (81.5)	26 (14.1)	5 (2.7)	3 (1.6)	0 (0.0)	176 (95.7)

a *χ_1_*2=27.8; *P*<.001.

## Discussion

In this quasi-experimental study conducted in real-world clinical settings, we demonstrated that a nurse-led, AI-assisted intelligent interactive platform significantly improved disease activity, functional status, medication adherence, and satisfaction in patients with RA over a 6-month follow-up period. Specifically, participants in the platform-based management group showed greater reductions in DAS28 scores and more substantial improvements in HAQ-II scores compared with those receiving routine postdischarge care. These findings highlight the potential of digital health interventions to enhance real-world RA management beyond conventional postdischarge care.

Our results align with and expand on evidence from large, multicenter studies showing that digital health apps can contribute to disease control in RA. In a pragmatic randomized clinical trial involving more than 2000 patients with RA, patients using a digital health app to assess patient-reported outcomes had higher rates of achieving a DAS28 based on C-reactive protein score of 3.2 or less at 6 months compared with controls, reflecting improved disease control with digital monitoring and clinician intervention [20]. Additionally, smaller prospective studies have reported clinically meaningful improvements in disease activity and other patient-reported outcomes following mHealth interventions for patients with inflammatory arthritis, underscoring the potential for mobile platforms to complement standard therapies [21]. There is systematic evidence further suggesting that asynchronous mHealth tools, including smartphone apps, web apps, and SMS text message reminders, are associated with desirable outcomes in RA self-management and health behaviors, although the heterogeneity of existing studies highlights the need for more rigorous designs [22]. Moreover, systematic reviews of digital health interventions for arthritis broadly support the role of these tools in enhancing medication adherence and quality of life, with most randomized controlled trials showing positive contributions of digital health in arthritis populations [23]. Together, these data contextualize our findings and indicate that digital tools, particularly those integrating real-time monitoring and clinician feedback, may improve RA outcomes beyond traditional follow-up models.

The mechanisms underlying observed improvements likely reflect multiple synergistic components of the platform. First, continuous data capture and AI-driven alerts enabled early identification of disease activity fluctuations, facilitating prompt clinical decision-making and adjustment of treatment plans consistent with treat-to-target strategies [24]. Second, personalized patient education modules and self-management support improved disease understanding, reduced anxiety-induced nonadherence, and reinforced positive health behaviors [25,26]. Third, integrated communication tools and nurse-led remote engagement fostered sustained patient-health care team interaction, thereby empowering patients and enhancing self-management [26-28], which are factors strongly associated with improved adherence in chronic disease management models. Collectively, this multimodal approach may underpin the greater improvements in functional status, symptom control, and engagement observed in the platform-based management group.

Importantly, enhanced functional outcomes (as measured using the HAQ-II) in the platform-based management group suggest that early and sustained control of inflammatory activity may translate into preserved musculoskeletal function and reduced disability progression. Remote access to rehabilitation guidance, individualized exercise recommendations, and rehabilitation education likely reduced care barriers for patients with mobility limitations or geographic challenges, expanding continuous care delivery beyond periodic clinic visits [14,25,28]. These effects are consistent with those observed in prior research [13,16] emphasizing the value of remote monitoring and patient engagement in optimizing long-term functional outcomes in chronic musculoskeletal diseases.

The platform significantly improved both medication adherence and patient satisfaction, which is consistent with findings from prior digital health interventions targeting chronic disease management [7,12,28]. Traditional outpatient follow-up is frequently constrained by logistical and geographical barriers, especially in rural or underserved areas, leading to delays in care and reduced compliance [9]. To address these challenges, the platform’s integrated “Medication Manager” provided automated medication reminders, individualized dosage guidance, and symptom reporting functions, thereby enabling timely clinical interventions in response to adverse events. Previous studies have provided evidence that such digital tools can decrease medication omissions and enhance treatment persistence in patients with RA and similar chronic conditions [10,23,29]. Moreover, the nurse-led, patient-centered platform empowered patients through shared decision-making, self-monitoring, and goal setting, which contributed to improved self-management. Personalized care plans and ongoing professional support not only reinforced adherence but also enhanced patient and nurse satisfaction with the care process [30,31]. These findings highlight the effectiveness of technology-assisted, nurse-led interventions in promoting satisfaction alongside adherence in remote care [32].

However, several limitations should be acknowledged. The quasi-experimental, campus-based allocation does not fully replicate randomized assignment, although this design reflects real-world implementation and enhances external validity. Participant and provider blinding was not feasible, which might introduce reporting bias; however, outcome assessors and analysts were blinded to mitigate this risk. The high-frequency data collected by the platform, such as daily pain and stiffness scores, were not included in this statistical analysis. Future research should leverage these real-time metrics to better characterize disease trajectories. A notable attrition rate was observed during the 6-month follow-up. Preliminary observations indicated that older patients with limited digital literacy or those with lower treatment compliance were more likely to drop out. These individuals represent a vulnerable population that might benefit the most from digital support but faces significant barriers to technology adoption. The lack of detailed demographics for these participants may limit the generalizability of our results regarding digital health equity. While the 6-month follow-up period was adequate to observe significant clinical improvements, longer follow-up is warranted to evaluate sustained disease control, radiographic progression, long-term disability outcomes, and cost-effectiveness. Future multicenter implementation studies with diverse patient populations, combined with qualitative research to explore user preferences, will be valuable to further validate and optimize digital care strategies for RA.

In conclusion, our study demonstrates that a nurse-led, AI-assisted intelligent interactive platform significantly improved clinical and patient-centered outcomes in postdischarge RA care. By facilitating continuous monitoring, timely clinical action, personalized education, and sustained engagement, this digital intervention offers a scalable model to enhance disease control and patient empowerment in routine rheumatology practice.

## Supplementary material

10.2196/90784 Multimedia Appendix 1 Intelligent interactive platform for rheumatoid arthritis (English- and Chinese-language versions of the web interface).

10.2196/90784 Multimedia Appendix 2 The integrated intelligent education module for rheumatoid arthritis (RA; partial interface in the Chinese-language version and all health education topics related to RA in the English-language version).

10.2196/90784 Multimedia Appendix 3 “Smart Medication Manager” in the platform for rheumatoid arthritis (English- and Chinese-language versions of the web interface).

10.2196/90784 Multimedia Appendix 4 The “Intelligent Consultation” module in the platform for rheumatoid arthritis (English- and Chinese-language versions of the web interface).

10.2196/90784 Checklist 1 STROBE checklist.

## Abbreviations

AI: artificial intelligence
DAS28: Disease Activity Score in 28 Joints
HAQ-II: Health Assessment Questionnaire–II
mHealth: mobile health
RA: rheumatoid arthritis
STROBE: Strengthening the Reporting of Observational Studies in Epidemiology
