# Supplementary material for: An Intelligent Interactive Management Platform for Rheumatoid Arthritis Care: Real-World Observational Study
Source: JMIR Med Inform. 2026 Apr 2;14:e90784. doi: 10.2196/90784 (PMC13046219; doi:10.2196/90784)
Supplement: Multimedia Appendix 2 [file medinform-v14-e90784-s002.docx]

**Multimedia Appendix 2:** The Integrated Intelligent Education Module for Rheumatoid Arthritis (Partial Interface in Chinese Version and All Health Education Topics Related to RA in English Version).


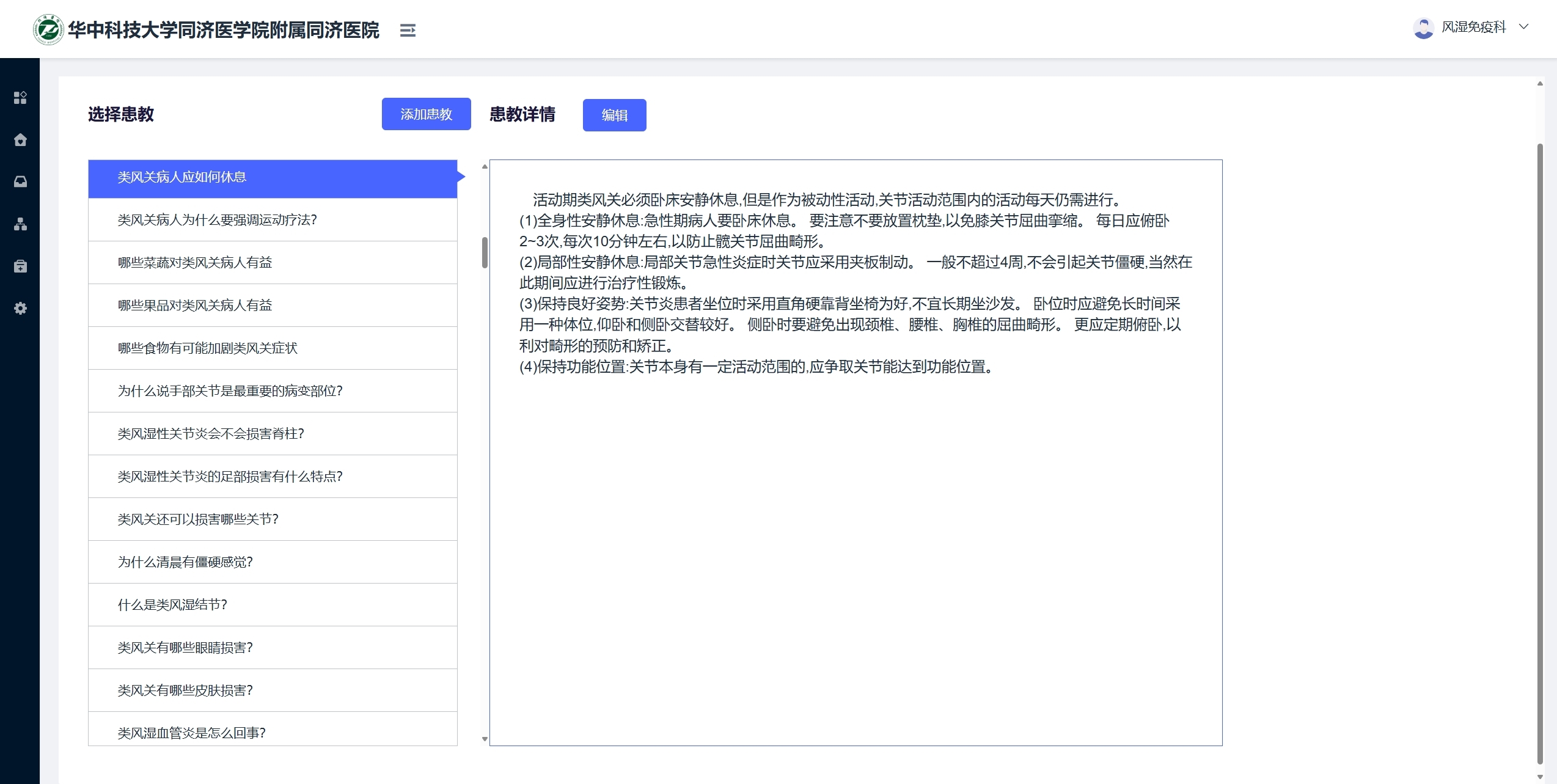


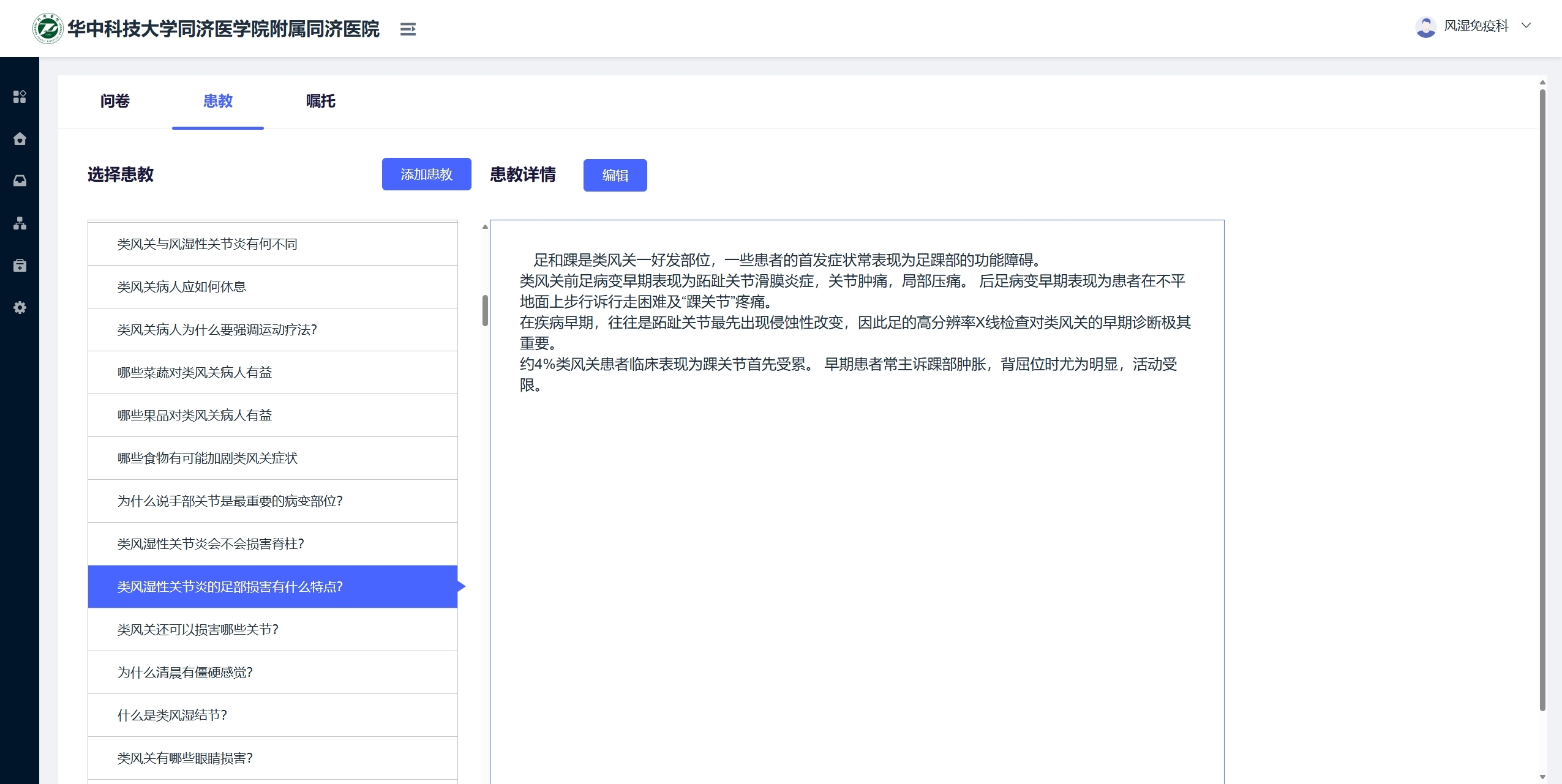


**Health Education Topics Related to RA in the Platform.**

| **1. Disease Overview and Pathophysiology**   - What is rheumatoid arthritis? - What are rheumatic and autoimmune diseases? - What are the causes of rheumatoid arthritis? - What is malignant rheumatoid arthritis? - Is rheumatoid arthritis associated with genetic factors? - Are rheumatic and autoimmune diseases hereditary? - Are rheumatic and autoimmune diseases truly incurable? - What is the difference between rheumatoid arthritis and rheumatic arthritis?   **2. Clinical Manifestations and Systemic Involvement**   - How is the range of joint motion assessed? - What are the clinical manifestations of rheumatoid arthritis? - Why are the hand joints considered the most important sites of involvement? - Can rheumatoid arthritis affect the spine? - What are the characteristics of foot involvement in rheumatoid arthritis? - Which other joints can be affected by rheumatoid arthritis? - Why does morning stiffness occur? - Morning stiffness: a warning sign of rheumatoid arthritis. - What is a rheumatoid nodule? - What types of ocular involvement can occur in rheumatoid arthritis? - What types of cutaneous manifestations can occur in rheumatoid arthritis? - Can rheumatoid arthritis affect the respiratory system? - Why should interstitial lung disease not be overlooked in patients with rheumatoid arthritis? - Does rheumatoid arthritis cause renal impairment? - Can rheumatoid arthritis affect the heart? - What types of skin rashes may indicate rheumatic or autoimmune diseases?   **3. Diagnosis and Prognosis**   - How is rheumatoid arthritis diagnosed? - How can rheumatoid arthritis be detected and diagnosed at an early stage? - Does a positive rheumatoid factor necessarily indicate rheumatoid arthritis? - What should be done when rheumatoid factor is found to be positive? - What are the early diagnostic autoantibody profiles for rheumatoid arthritis, and what is their clinical significance? - What is undifferentiated arthritis? - What is seronegative rheumatoid arthritis? - What is elderly-onset rheumatoid arthritis? - What is juvenile rheumatoid arthritis (JRA)? - Why are multiple diagnostic tests necessary for rheumatoid arthritis? - How can the prognosis of rheumatoid arthritis be evaluated?   **4. Treatment and Medication Management**   - What are the principles of treatment for rheumatoid arthritis? - Is pain relief alone sufficient for the treatment of rheumatoid arthritis? - Why is individualized treatment particularly emphasized in rheumatic and autoimmune diseases? - What is the mechanism of action of leflunomide, and what are its side effects? - What is methotrexate, and how can it be used safely? - What are the side effects of methotrexate? - What precautions should be taken during methotrexate therapy? - Can pregnant women use hydroxychloroquine long-term? - How should hydroxychloroquine be taken correctly? - Can biological agents provide a definitive cure? - Can plasma exchange treat rheumatic and autoimmune diseases, and what issues should be considered? - Can dialysis remove rheumatoid factor or treat rheumatoid arthritis?   **5. Rehabilitation, Exercise, and Lifestyle Management**   - How should patients with rheumatoid arthritis plan rest and physical activity? - How should patients with rheumatoid arthritis arrange rest appropriately? - Why is exercise therapy emphasized for patients with rheumatoid arthritis? - Joint Exercise Video *(Standard Definition)* - Joint Exercise Video *(High Definition)* - How should patients with arthritis undergo rehabilitation therapy? - How can patients with rheumatic and autoimmune diseases select appropriate rehabilitation therapies? - What are the rehabilitation measures for patients with rheumatoid arthritis? - What dietary considerations should patients with rheumatoid arthritis keep in mind? - Which vegetables are beneficial for patients with rheumatoid arthritis? - Which fruits are beneficial for patients with rheumatoid arthritis? - Which foods may aggravate the symptoms of rheumatoid arthritis? - What should I do if my joints are painful or inflamed? - How should patients with rheumatoid arthritis perform personal self-care? - Can patients with rheumatic or autoimmune diseases receive cupping therapy? - Why should patients with rheumatic and autoimmune diseases avoid smoking?   **6. Special Populations and Life Events**   - How does pregnancy influence rheumatoid arthritis? - Can children develop rheumatoid arthritis? - How should juvenile rheumatoid arthritis be managed? - What are the clinical characteristics of rheumatoid arthritis in the elderly? - How should elderly-onset rheumatoid arthritis be appropriately treated? - Why are elderly individuals more prone to falls? - How can older adults prevent falls? - Why do many patients with rheumatic and autoimmune diseases feel sensitive to cold, especially in their joints? - Why are some patients with rheumatic and autoimmune diseases prone to catching colds? - Why are some patients with rheumatic and autoimmune diseases sensitive to sunlight? - Why do patients with rheumatic and autoimmune diseases often experience fatigue? - Why can menstruation, miscarriage, or childbirth trigger or exacerbate rheumatic and autoimmune diseases?   **7. Self-Management, Follow-up, and Psychological Care**   - Why is it important to seek early consultation with a rheumatology specialist? - Why should patients with rheumatoid arthritis maintain frequent communication with their rheumatology specialists? - How should patients with rheumatoid arthritis schedule follow-up visits? - What should patients with rheumatoid arthritis pay attention to after hospital discharge? - How should patients with rheumatic and autoimmune diseases manage their medical records effectively? - What are the common psychological problems among patients with rheumatic and autoimmune diseases? - How can patients cope with rheumatic and autoimmune diseases from a psychological perspective? |
| --- |
