# Supplementary material for: An Intelligent Interactive Management Platform for Rheumatoid Arthritis Care: Real-World Observational Study
Source: JMIR Med Inform. 2026 Apr 2;14:e90784. doi: 10.2196/90784 (PMC13046219; doi:10.2196/90784)

**Multimedia Appendix 3:** Smart Medication Manager in Platform for Rheumatoid Arthritis (English and Chinese Versions of the Web Interface).


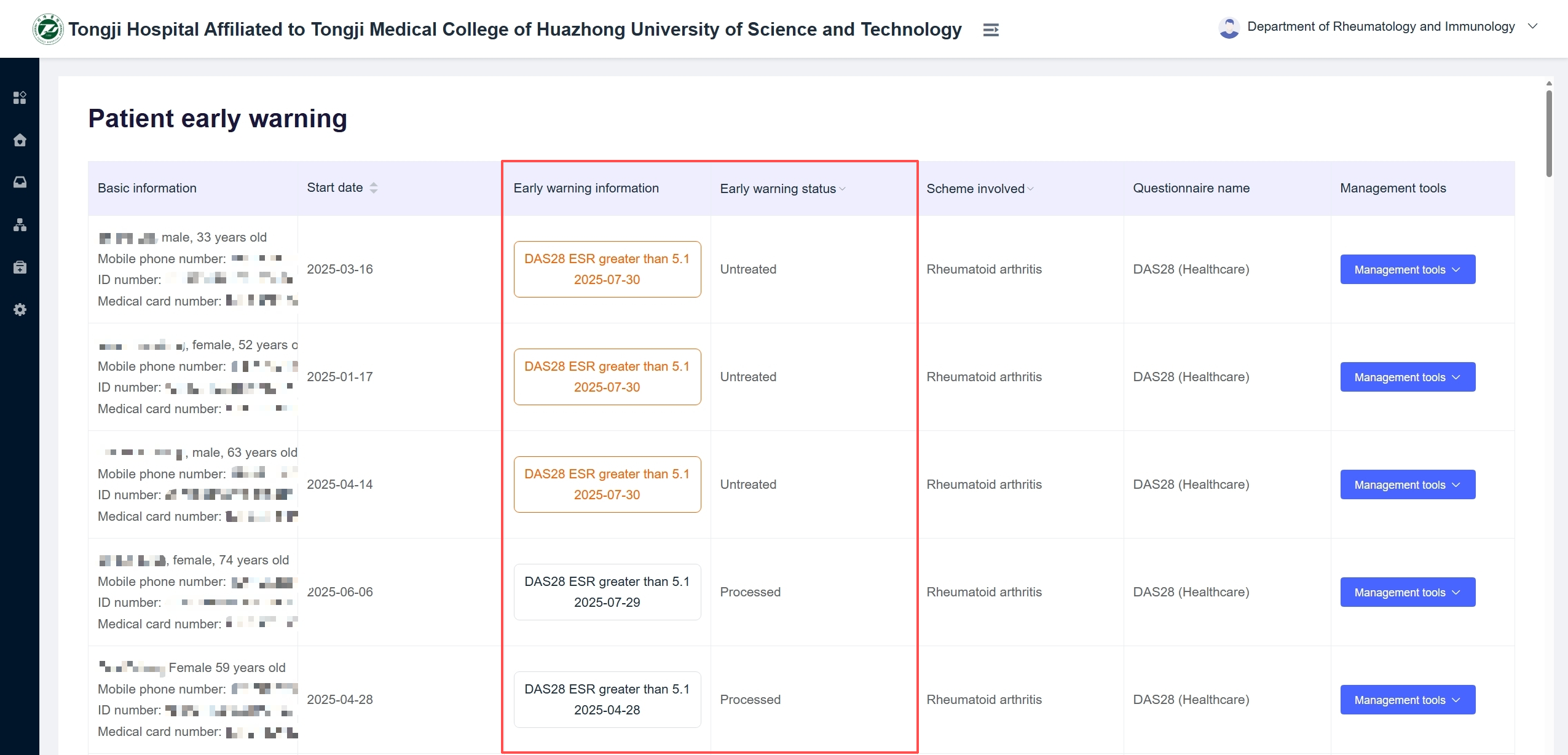


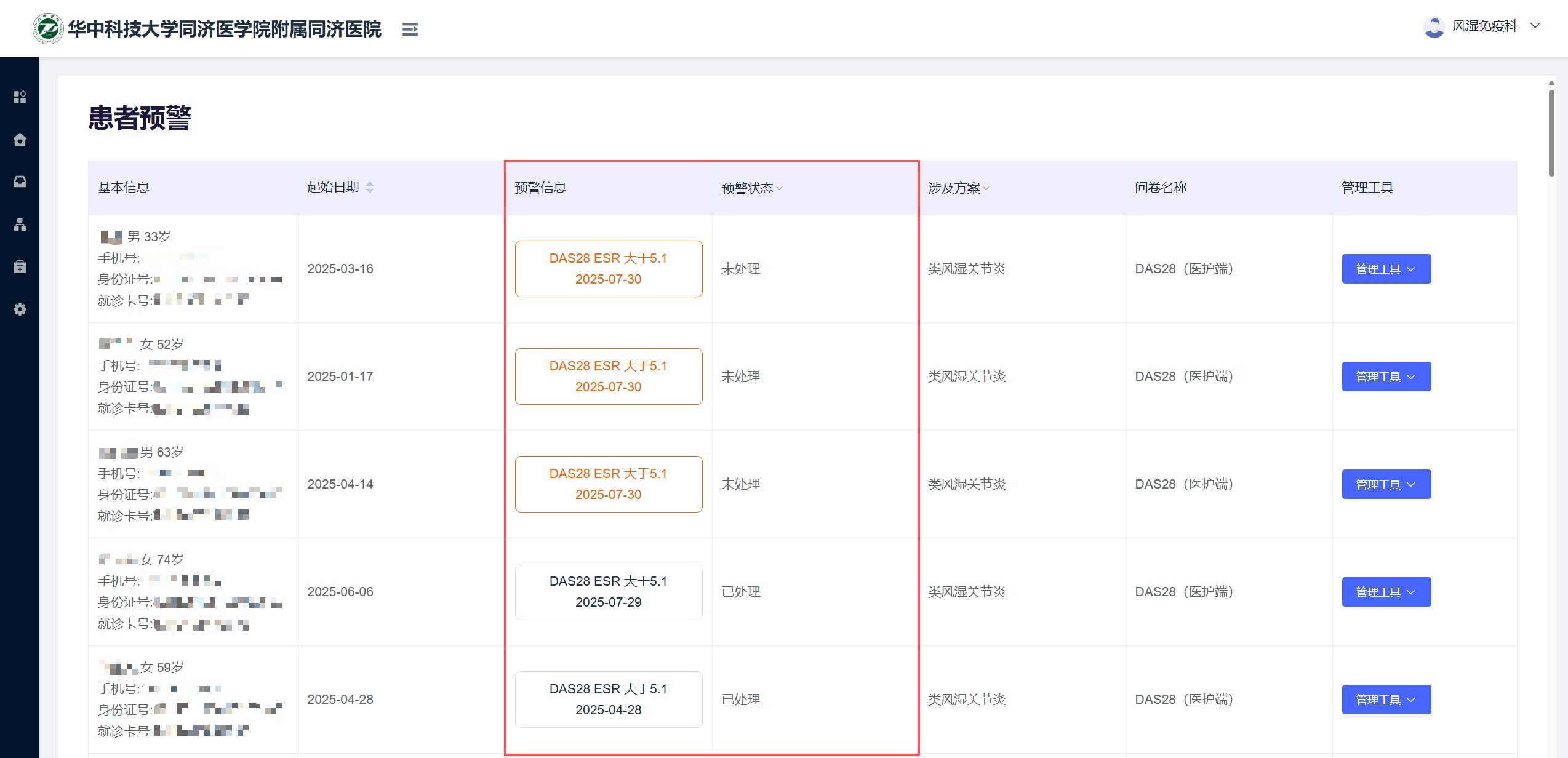


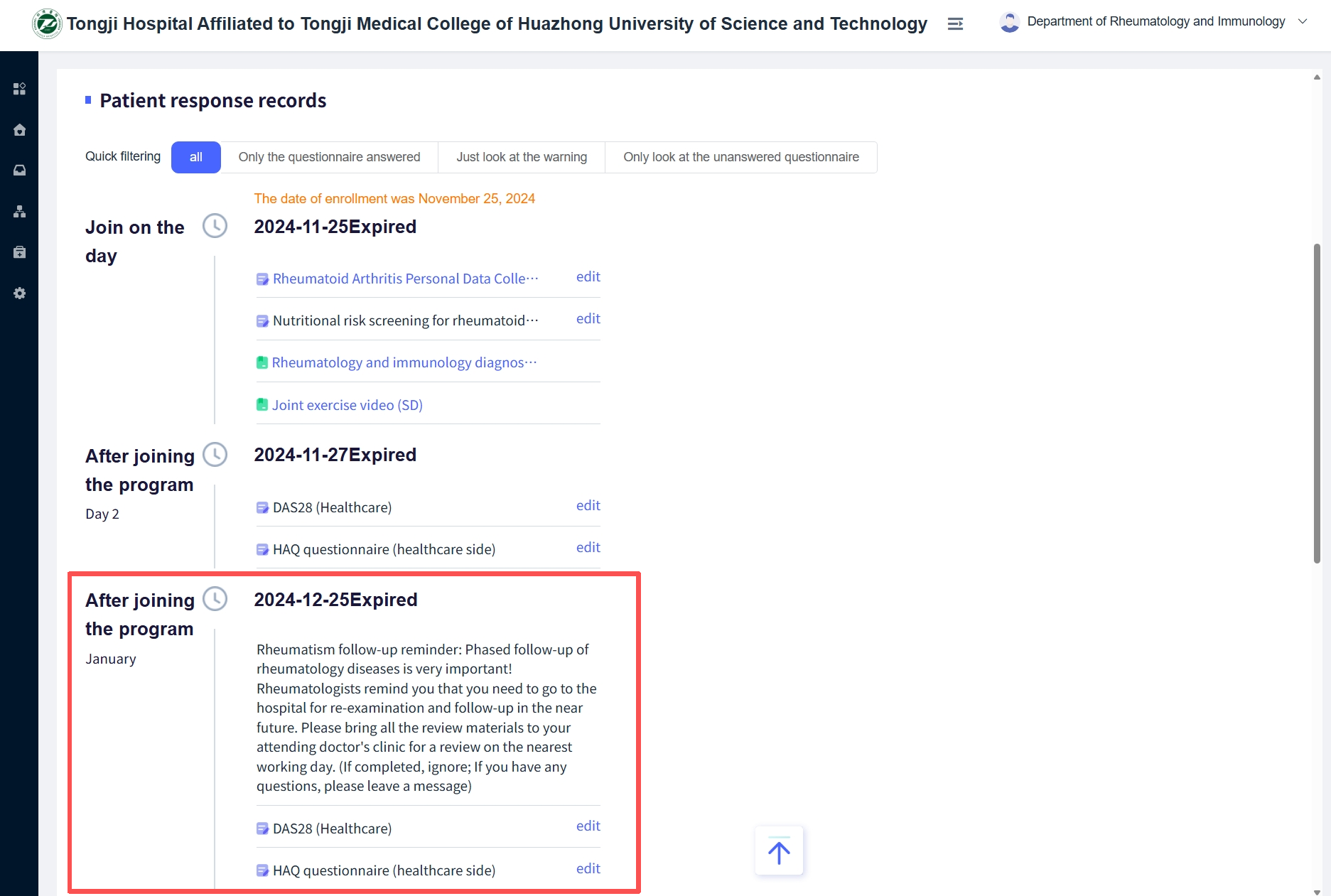


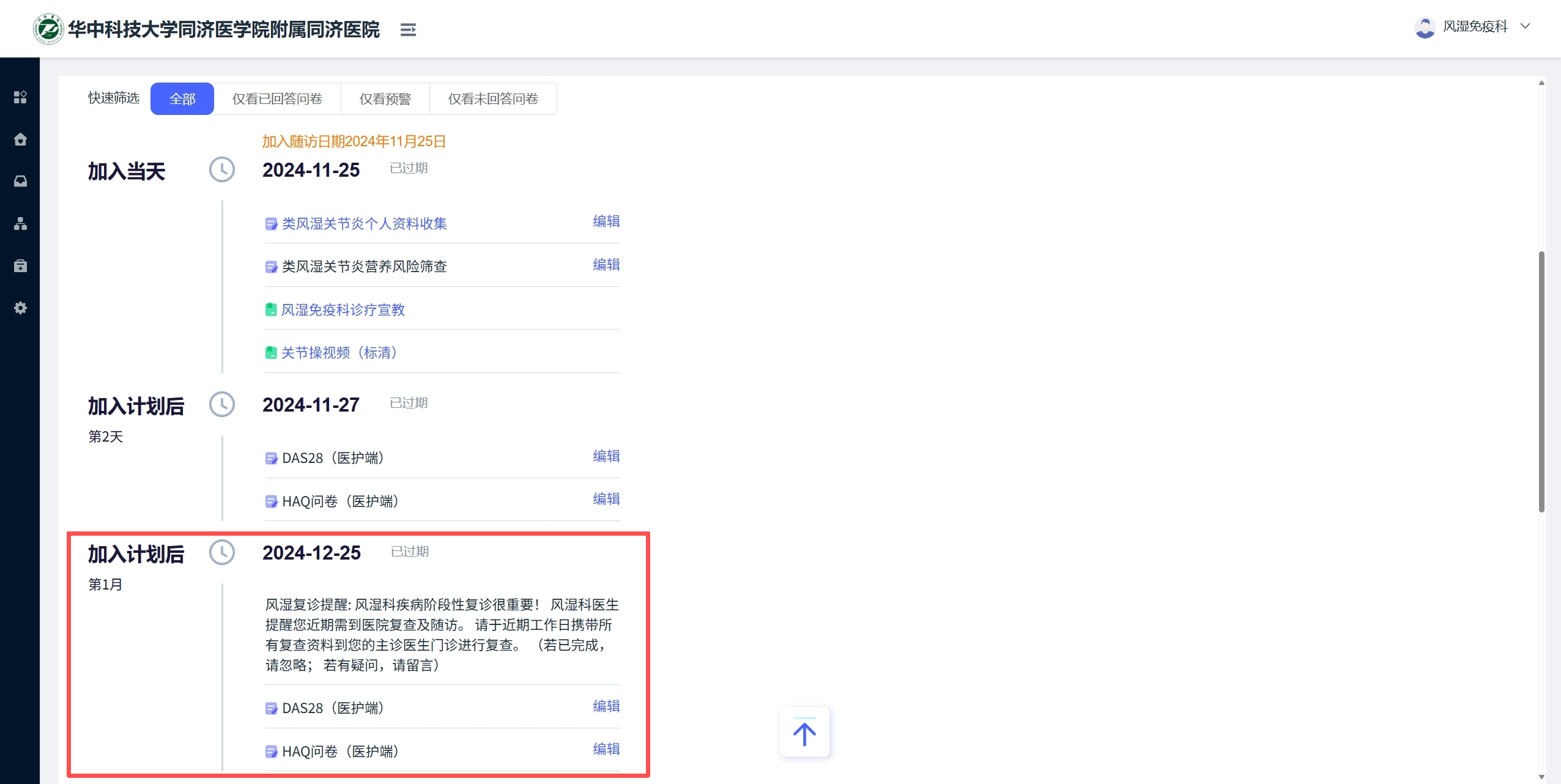


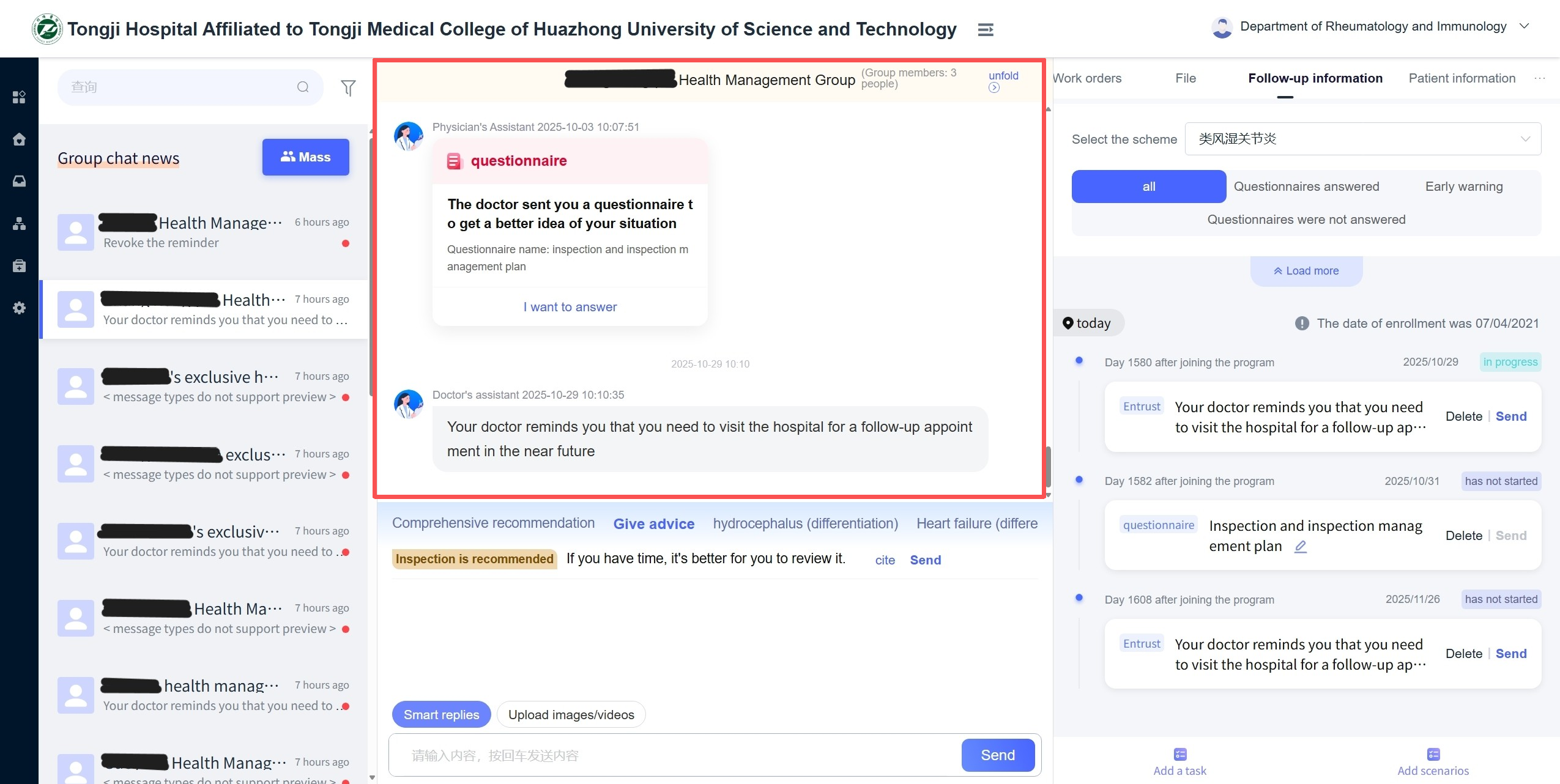


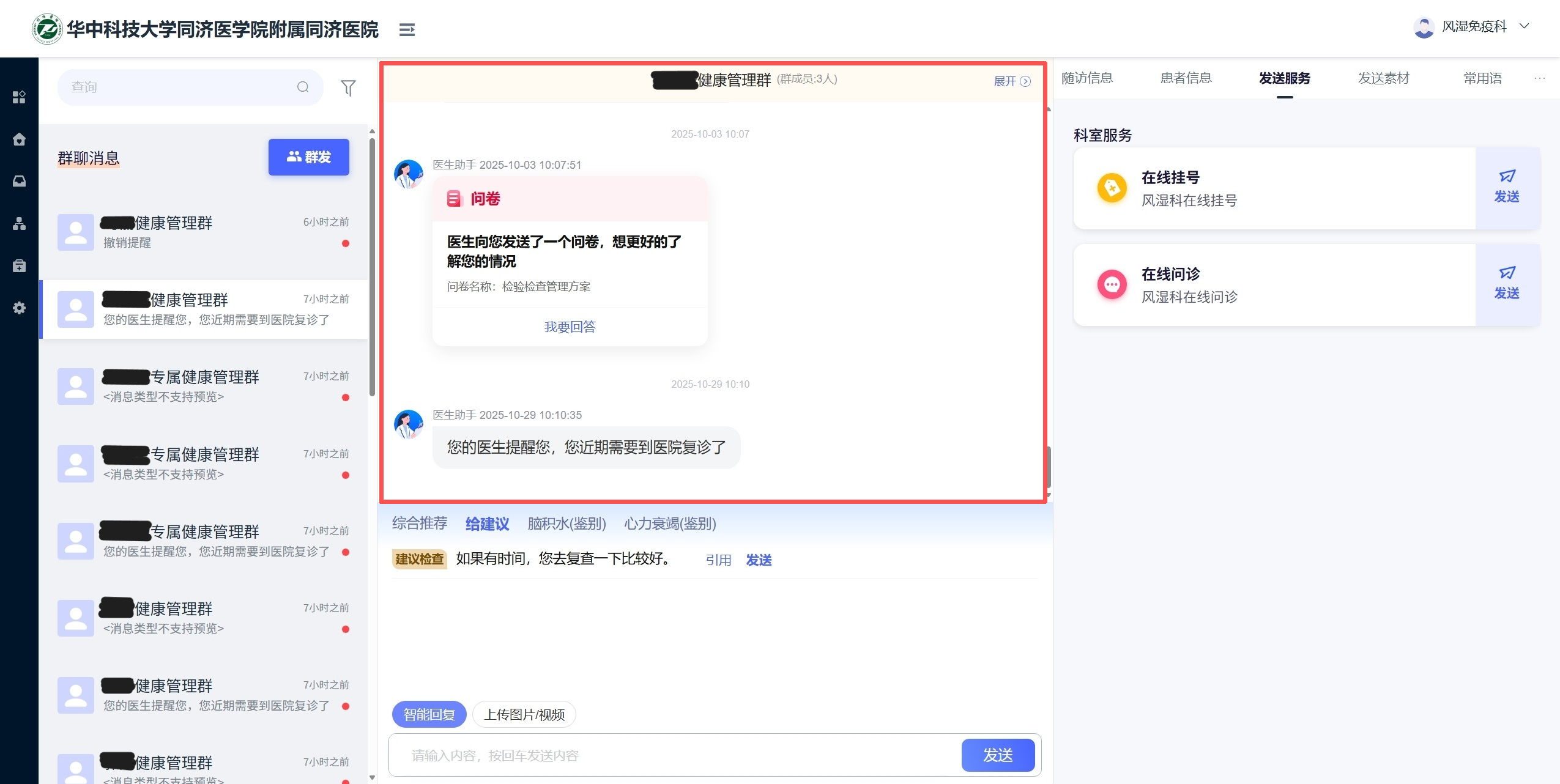

Supplement: Multimedia Appendix 3 [file medinform-v14-e90784-s003.docx]
