# Supplementary material for: An Intelligent Interactive Management Platform for Rheumatoid Arthritis Care: Real-World Observational Study
Source: JMIR Med Inform. 2026 Apr 2;14:e90784. doi: 10.2196/90784 (PMC13046219; doi:10.2196/90784)

**Multimedia Appendix 4:** The “Intelligent Consultation” module in Platform for Rheumatoid Arthritis (English and Chinese Versions of the Web Interface).


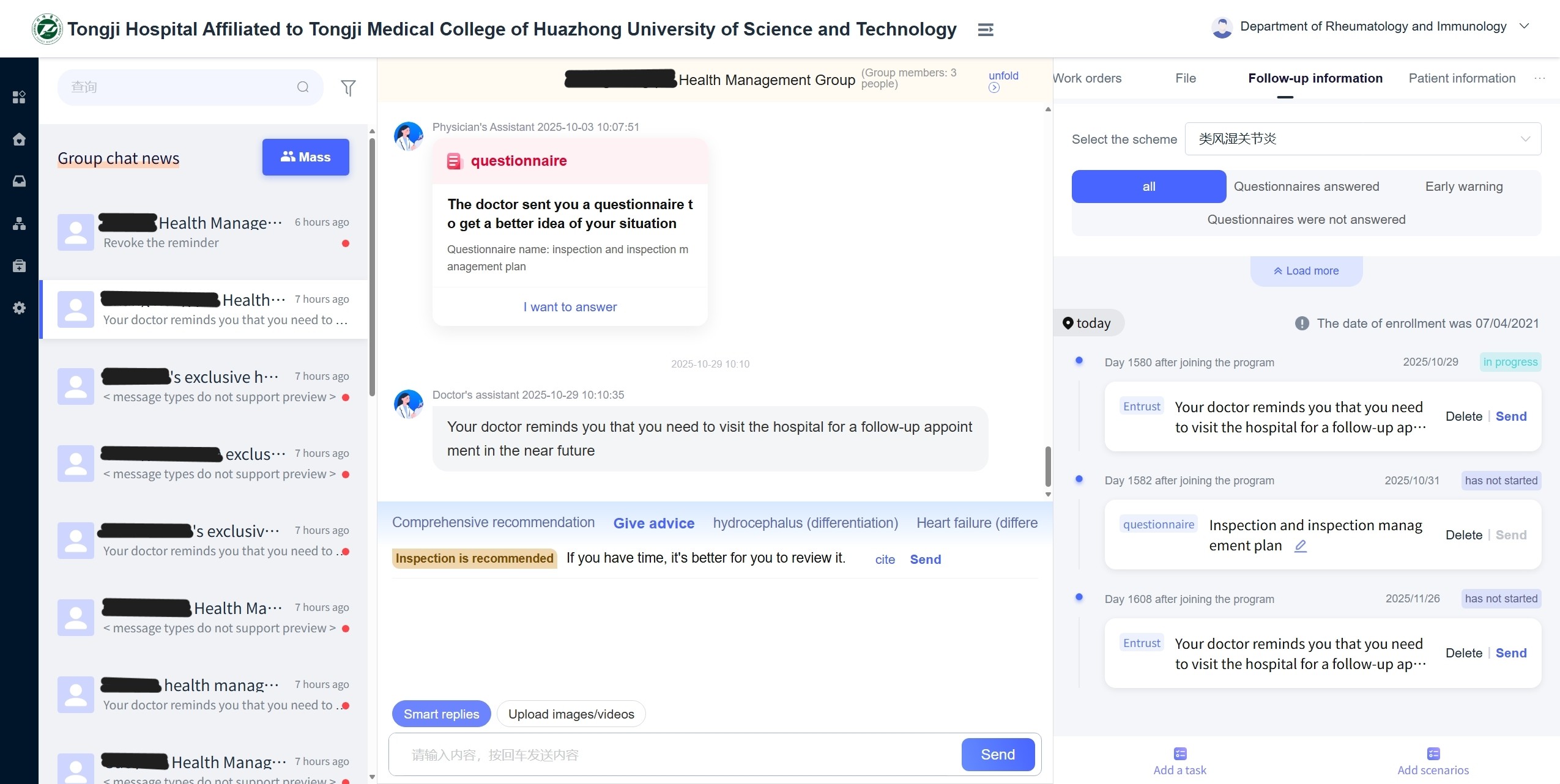


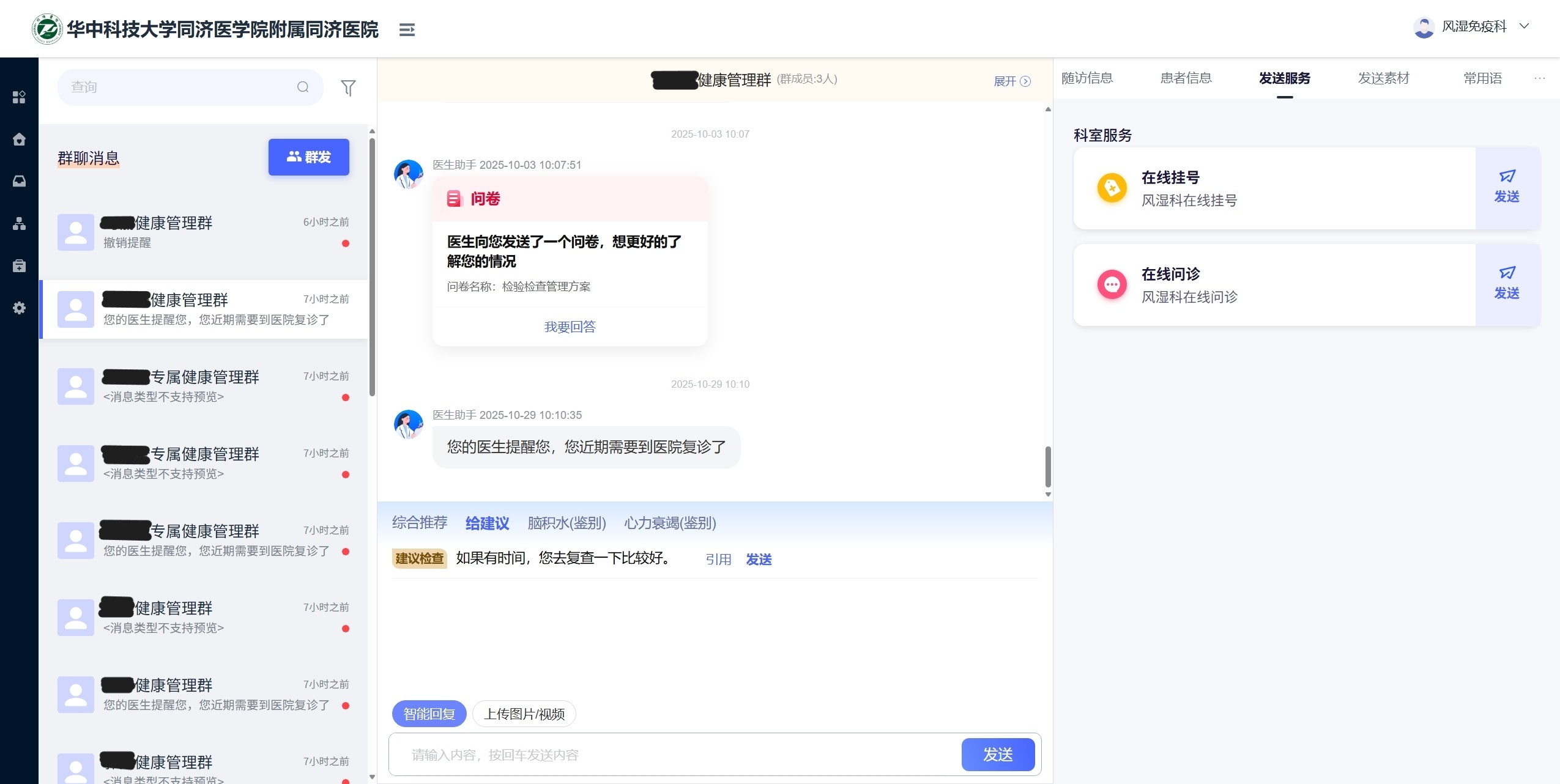


**Previous Chat Records on Mobile Device (English and Chinese Versions).**


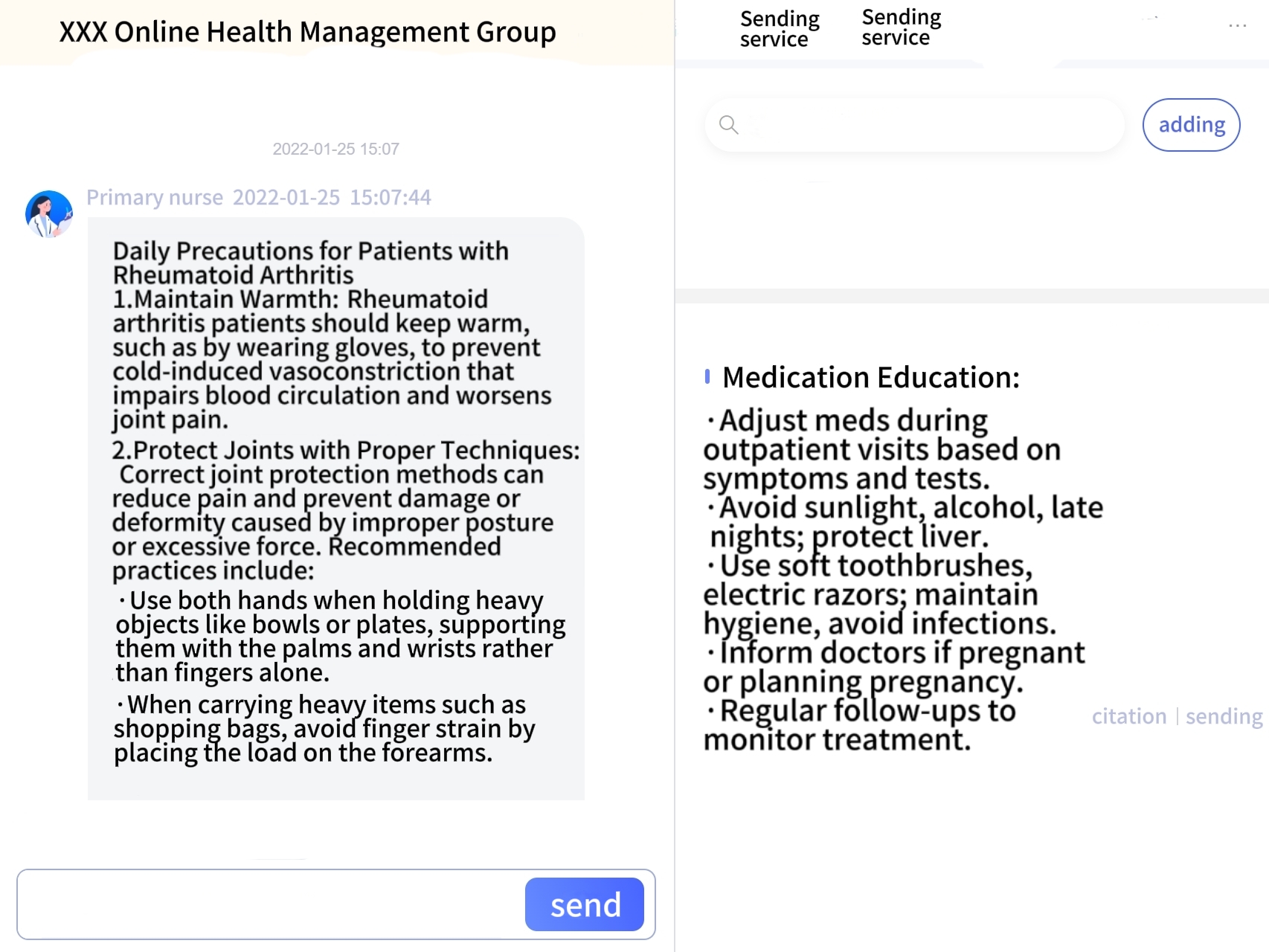


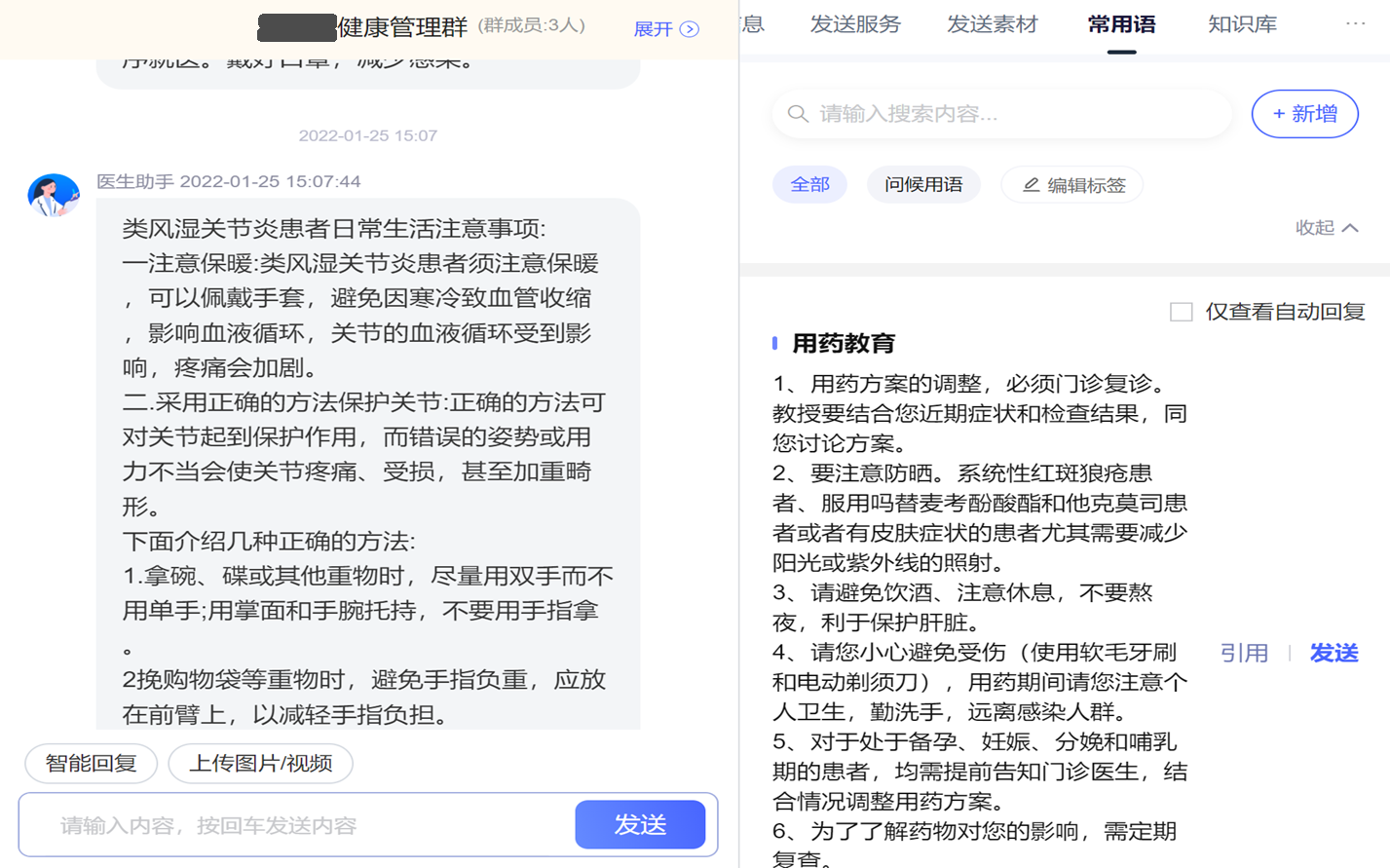

Supplement: Multimedia Appendix 4 [file medinform-v14-e90784-s004.docx]
